# Editorial: The regulation of proteostasis in aging

**DOI:** 10.3389/fcell.2023.1221510

**Published:** 2023-05-25

**Authors:** Ji-Xin Tang, Fu-Hui Xiao

**Affiliations:** ^1^ Shunde Women and Children’s Hospital, Guangdong Medical University (Foshan Shunde Maternal and Child Healthcare Hospital), Foshan, China; ^2^ Guangdong Provincial Key Laboratory of Autophagy and Major Chronic Non-communicable Diseases, Institute of Nephrology, Affiliated Hospital of Guangdong Medical University, Zhanjiang, China; ^3^ Kunming Institute of Zoology, Chinese Academy of Sciences, Kunming, China

**Keywords:** aging, proteostasis, non-canonical autophagy, autophagic flux, proteasome, protein translation

## Introduction

Aging is regarded as a biological process characterized by the progressive loss of physiological integrity, leading to increased risk for many chronic diseases, such as diabetes, cancer, kidney diseases, cardiovascular disorders, and neurodegenerative diseases (López-Otín et al., 2013; Cai et al., 2022). As the population ages rapidly, age-related chronic diseases have been becoming the major risk factors to human health and survival. Therefore, a better understanding of the mechanism of aging would be able to improve the quality of life of older people.

Protein homeostasis or proteostasis is a dynamic process in which mammalian cells can keep the balance of proteome by regulating protein synthesis, folding, transport, post-translational modification, and degradation. The loss of proteostasis is a hallmark of aging and may be a primary cause of aging (López-Otín et al., 2023). Proteostasis is controlled by the proteostasis network, including chaperones, ubiquitin-proteasome and lysosomal-autophagy systems, which maintain the stability and function of the cellular proteome through preventing the accumulation of aggregated or damaged proteins (Liang et al., 2022a). In addition, a large body of evidence supports a close relationship between disturbed proteostasis and aging and age-related diseases (Cai et al., 2022). Thus, understanding the regulation of proteostasis in aging is essential to develop new strategies to extend lifespan and healthspan in humans. The 5 articles in this Research Topic, including 3 reviews, 1 mini review and 1 method, described the association between proteostasis and aging in several critical aspects.

## Proteostasis in aging and cancer

Aging promotes a range of degenerative diseases characterized by a debilitating loss of tissue or cell function in almost all the species. Meanwhile, aging can also promote proliferative disorders, among which the most deadly disease is cancer. Chen et al. summarized the regulatory mechanisms of proteostasis in mammalian cells and discussed the function of proteostasis in aging and cancer (Chen et al.). In this review, they proposed an important point worth considering: interventions to maintain protein homeostasis may promote longevity and healthy life in mammals, but may also lead to an increased risk of cancer.

The genome of cancer cells is filled with various genetic mutations, leading to the production of misfolded proteins. Therefore, cancer cells need to rely heavily on protein quality control network to maintain protein homeostasis and the uncontrolled growth. Therefore, when we extend the lifespan of mammals (even human) by enhancing protein homeostasis regulatory networks such as enhanced autophagy, proteasome pathways, or overexpressed chaperones, it may increase an individual’s risk of cancer. Therefore, we need to further investigate the role of protein homeostasis in aging and cancer, in order to develop strategies that can both extend personal lifespan and prevent cancer occurrence.

## The proteasome, a key modulator of proteostasis, in brain aging and neurodegenerative disease

As a multi-subunit proteolytic complex, proteasome play an essential role in the maintenance of mammalian cellular proteostasis by degrading abnormal proteins through multiple ubiquitin molecules. Davidson et al. systematically reviewed the role and mechanism of proteasome, a key factor to maintain protein homeostasis, in brain aging and neurodegenerative diseases (Davidson and Pickering). At present, a large number of studies have shown that the function of the proteasome is decreased during aging and aging-related diseases, however it is still unclear whether the decreased proteasomal function leads to the corresponding outcomes through the loss of proteostasis. In addition, the reasons for the age-related decline in proteasome function still need to be further clarified.

## Non-canonical autophagy in aging and age-related diseases

As an evolutionarily conserved cellular process, autophagy is another essential mechanism to maintain mammalian cellular proteostasis through degrading protein aggregates via lysosomes (Liang et al., 2022b). Loss of proteostasis and disabled macroautophagy are the two hallmarks of aging (López-Otín et al., 2023). In this Research Topic, Kumar and Mills reviewed the non-canonical autophagy, a special class of autophagy processes that can be either degradative or secretory (Kumar and Mills). Like classical autophagy, the dysfunction of non-classical autophagy is always observed in aging and age-related diseases. However, how these abnormalities function in aging and aging-related diseases remain to be investigated deeply. In especial, one most important thing, as Kumar and Mills pointed out, would be how we can use non-classical autophagy to prevent, diagnose and even treat aging and age-related diseases (Kumar and Mills).

## Protein translation paradox in aging

Mammalian intracellular proteostasis is not only regulated by intracellular proteostasis network, but also influenced by other factors, such as protein translation. It is known that the protein translation decreases gradually with age in different organisms, indicating its driving role in aging and age-related diseases by leading to the imbalance of proteostasis. For example, a recent study showed that aging can cause ribosome pausing and thus lead to the proteostasis collapse (Stein et al., 2022). However, amounting evidence has shown that inhibiting protein translation *in vivo* through genetic intervention or the use of small molecule inhibitors can promote proteostasis and inhibit the progression of aging and age-related diseases. For example, Xiao et al. showed that human healthy aging can be regulated by ETS1 through decreasing ribosomal activity (Xiao et al., 2022). Here, Kim and Pickering discussed the paradox of protein translation in aging and longevity and highlighted the significance of fully understanding the mechanisms of translational regulation in aging (Davidson and Pickering).

## The autophagic flux in peripheral blood mononuclear cells

In this Research Topic, Walter et al. also introduced two methods to determinate the autophagic flux in murine and human peripheral blood mononuclear cells (Walter et al.). As mentioned above, autophagy is a key process to maintain cellular proteostasis. Therefore, evaluating the autophagy flux in peripheral blood mononuclear cells may provide an approach to analyze the proteostasis of organisms, especially humans.
